# A Facial Strategy for Catalyst and Reducing Agent Synchronous Separation for AGET ATRP Using Thiol-Grafted Cellulose Paper as Reducing Agent

**DOI:** 10.3390/polym10010026

**Published:** 2017-12-25

**Authors:** Xiaowu Jiang, Jie Han, Lunan Cao, Yan Bao, Jian Shi, Jing Zhang, Lingli Ni, Jing Chen

**Affiliations:** Jiangsu Provincial Key Laboratory of Palygorskite Science and Applied Technology, College of Chemical Engineering, Huaiyin Institute of Technology, Huai’an 223003, China; han199726jie@163.com (J.H.); caolunan920@163.com (L.C.); 1525388132@163.com (Y.B.); shijian873686067@163.com (J.S.); zhangjing860321@hyit.edu.cn (J.Z.); jingchen@hyit.edu.cn (J.C.)

**Keywords:** AGET ATRP, thiol-functionalized cellulose paper, synchronous separation, metal catalyst and byproduct of reducing agent

## Abstract

Atom Transfer Radical Polymerization (ATRP) has been a powerful tool to synthesize well-defined functional polymers, which are widely used in biology, drug/gene delivery and antibacterial materials, etc. However, the potential toxic residues in polymer reduced its service life and limited its applications. In order to overcome the problem, in this work, a novel polymerization system of activators generated by electron transfer for atom transfer radical polymerization (AGET ATRP) for synchronous separation of the metal catalyst and byproduct of reducing agent was developed, using thiol-grafted cellulose paper (Cell-SH) as a solid reducing agent. The polymerization kinetics were investigated in detail, and the “living” features of the novel polymerization system were confirmed by chain-end analysis and chain extension experiment for the resultant polymethyl methacrylate (PMMA). It is noted that the copper residual in obtained PMMA was less than 20 ppm, just by filtering the sheet-like byproduct of the reducing agent.

## 1. Introduction

Atom Transfer Radical Polymerization (ATRP) [1,2,3,4] has been a powerful tool to synthesize different functional polymers with controlled molecular weight and precise topological structures, such as block polymer, cyclic polymer, star polymer and hyperbranched polymer, which are widely used in biology, drug/gene delivery and antibacterial materials, etc. However, normal ATRP was catalyzed by lower oxidation state metal catalyst [5,6,7,8], which is susceptible to oxygen; therefor, the process of catalyst complex storing and handling could be challenging. Fortunately, Matyjaszewski’s group developed a series of stable high valence metal catalyzed ATRPs [9,10]; among them, activator generated by electron transfer for ATRP (AGET ATRP) [11,12,13,14] was the most popular system because of the ease of operation and applicability. As a multicomponent polymerization system, a large amount of metal catalyst and reducing agent was used in AGET ATRP. Therefore, the obtained controlled polymer always had much impurity. The potential toxic residues in polymer reduced its service life and limited its applications, especially in biology.

In fact, some efficient ways have been developed for reducing the poisonous metal complex in obtained polymer, that is, (1) developed highly active catalyzed system to reduce the usage of metal catalyst to ppm level [15,16,17,18], but excess of ligand and reducing agent were necessary in this system, which always remained in obtained polymers; (2) developed metal catalyst recycled systems: solid-supported catalyst system (solid-supported catalysis by covalent bonding [19,20,21,22,23,24], soluble polymer supported catalysts [25], immobilized/soluble hybrid system [26] and reversible supported catalysts [27]) and liquid/liquid biphasic system (ionic liquids biphasic system [28,29], fluorous biphasic system [30], aqueous/organic biphasic system [31,32,33] and thermomorphic polar/nonpolar organic biphasic system [34,35]) to efficiently separate and recycle metal catalyst; however, the concerns focused on the metal catalyst, regardless of other contamination in polymers; (3) developed metal free ATRP [36,37,38,39,40] with organic molecular instead of metal catalyst. Nevertheless, how to avoid the disadvantage of organic matter is a question. As shown above, although the metal catalyst with relative high toxicity in polymers could almost be wiped out, what about others? A strong electrophilic byproduct of the reducing agent, such as dehydroascorbate [41], has been reported to react with protein chains and change the chemical structure of DNA. So, how to efficiently and simply remove metal catalyst and reducing agent from AGET ATRP to obtain a pure well-defined polymer is an urgent issue.

Recently, Felpin’s group developed a biomimetic reducing agent, thiol functionalized cellulose paper (Cell-SH), for the highly selective reduction of Cu^II^ to Cu^I^ with simultaneous detection of copper by the adsorption of the copper catalyst and a change of color [42]. Moreover, Cell-SH can be used as an efficient heterogeneous reducing agent and copper adsorbent in copper catalyzed click chemistry [43]. By optimizing the reaction conditions, the copper adsorption efficiency reached 97.5%. 

**Scheme 1:**
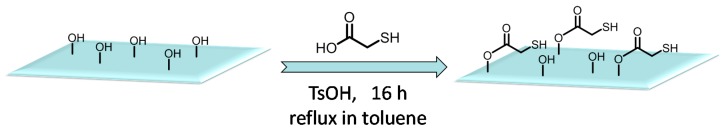
Synthetic pathway of reducing agent, Cell-SH.

Therefore, in this paper, we aimed at establishing a novel system for metal catalyst and byproduct synchronous separation of AGET ATRP with Cell-SH paper as dual tool, promoting the Cu catalyzed AGET ATRP through the reduction of Cu^II^ to Cu^I^ and adsorbing copper catalyst from polymer. Herein, in this system, Cell-SH (Scheme 1) was synthesized as reducing agent, copper(II) bromide (CuBr_2_) was employed as metal salt, *N*,*N*,*N*′,*N*″,*N*″-Pentamethyldiethylenetriamine (PMDETA) was optimized as ligand, methyl methacrylate (MMA) was chosen as model monomer, ethyl-2-bromo-2-phenyl acetate (EBPA) was selected as initiator. The thiols grafted on the cellulose paper reduced the Cu(II) complex to Cu(I) complex during the AGET ATRP, meanwhile, the thiols were oxidized to disulfide compounds, which efficiently adsorbed the copper complex. After the polymerization, the cellulose paper adsorbed with metal catalyst was separated easily by filtration, so that, the clean and well-defined polymer was obtained in situ.

## 2. Experiment Section

### 2.1. Materials

Methyl methacrylate (MMA, >99%) was purchased from shanghai Chemical Reagents Co. Ltd. (Shanghai, China), and the inhibitor was removed by passing through a neutral alumina column before use. Copper bromide (CuBr_2_, >99%) was purchased from shanghai Chemical Reagents Co. Ltd. (Shanghai, China), and used as received. *N*,*N*,*N*′,*N*″,*N*″-Pentamethyldiethylenetriamine (PMDETA), Thioglycolic acid and *p*-methylbenzenesulfonic acid were purchased from J&K Scientific Ltd. (Shanghai, China), and used as received. Ethyl-2-bromo-2-phenyl acetate (EBPA, 97%) was purchased from Alfa Aesar (Tianjin, China), and used as received. Whatman^®^ grade 6 paper (100 mm Ø) (Bitai Bio-Technique Co. Ltd., Shanghai, China) was the source of cellulose paper. Sodium hydroxide (99%), ethanol (>99.5%), toluene (>99.5%), acetone (>99.5%), dichloromethane (>99.5%), tetrahydrofuran (THF, >99.5%), methanol (>99.5%) and ethyl acetate (>99.5%) were purchased from ChinaSun Specialty Products Co. Ltd. (Suzhou, China), and used as received.

### 2.2. Synthesis of Biomimetic Reducing Agent, Thiol Functionalized Cellulose Paper (Cell-SH)

The synthetic procedure of Cell-SH was similar to the previous literature [37] and as followed: Firstly, the cellulose paper was pretreated by aqueous sodium hydroxide: 5 g cellulose paper was immersed in 400 mL aqueous sodium hydroxide (40 g, 10%) and stirred gently over night. Then the obtained cellulose paper was washed 3 times by ethanol and stored in ethanol. Secondly, the pretreated cellulose paper was functionalized by thioglycolic acid: Thioglycolic acid (2.5 mL) and catalytic *p*-methylbenzenesulfonic acid (250 mg) was added to 200 mL toluene, then 2 g cellulose paper was tailored into appropriate size and added to the solution. The mixture was refluxed for 16 h under Ar, then cooled down to room temperature. The functionalized cellulose paper was in turn washed by methanol, ethanol, acetone and dichloromethane under sonication bath for several minutes, dried at 30 °C under vacuum, and stored under Ar Elemental Analysis: C: 41.62%, H: 5.23%, S: 7.98%*,* found from a Vario ELcube elemental analysis instrument (Elementar Trading Shanghai Co. Ltd., Shanghai, China). 

### 2.3. General Procedure of AGET ATRP of MMA with Cell-SH Paper as Reducing Agent

A typical polymerization procedure for the molar ratio of [MMA]_0_/[EBPA]_0_/[CuBr_2_]_0_/[PMDETA]_0_ = 400/1/0.25/0.5 was as follows: a mixture was obtained by adding MMA (2.0 mL, 18.8 mmol), EBPA (8.2 μL, 47.4 × 10^−3^ mmol), CuBr_2_ (2.7 mg, 11.8 × 10^−3^ mmol), PMDETA (4.9 μL, 23.7 × 10^−3^ mmol), anisole (1.0 mL) to a dried ampoule with a stir bar, then 50 mg Cell-SH was mixed into the ampoule as the reducing agent. The ampoule was sealed by flame and transferred to an oil bath with the temperature of 90 °C. The polymerization conduct with stirring for designed time. And the ampoule was transferred to ice water to cool down to room temperature, then unfolded. THF (~2 mL) was added to the mixture to dilute the solution. The oxidized Cell-SH paper was separated by filtration, and the filtrate was precipitated into a large amount of methanol (~200 mL). The polymer obtained by filtration was dried under vacuum until constant weight at 30 °C. The monomer conversions were determined from the mass ratio of added monomer to obtained polymer. 

### 2.4. Chain Extension with PMMA as the Macroinitiator

A predetermined quantity of PMMA was added into a dried ampoule, and then the predetermined quantity of MMA, CuBr_2_, PMDETA and anisole were added with the molar ratio of [MMA]_0_/[PMMA]_0_/(CuBr_2_]_0_/[PMDETA]_0_ = 400/2/0.25/0.5, then 50 mg Cell-SH paper was added to the mixture as reducing agent. The ampoule flame-sealed directly and then transferred into an oil bath held by a thermostat at the desired temperature (90 °C) to polymerize under stirring. The rest of the procedure was the same as the AGET ATRP of MMA with Cell-SH paper as reducing agent described above.

### 2.5. Characterization

The values of the number-average molecular weight (*M*_n, GPC_) and molecular weight distribution (*M*_w_/*M*_n_) of PMMA were obtained from a TOSOH HLC-8320 gel permeation chromatograph (GPC) (TOSOH Bioscience Shanghai Co. Ltd., Shanghai, China), which equipped with a TOSOH refractive-index detector, using 4.6 × 20 mm guardcolumn (TSKgel SuperMP-N, TOSOH Bioscience Shanghai Co. Ltd., Shanghai, China) and two 4.6 × 150 mm detector column (TSKgel SupermultiporeHZ-N, TOSOH Bioscience Shanghai Co. Ltd., Shanghai, China) with measurable molecular weight ranging from 500 to 5 × 105 g·mol^−1^. THF was used as the eluent with a flow rate of 0.35 mL/min and temperature of 40 °C. GPC samples were injected by autosampler (TOSOH plus, company, TOSOH Bioscience Shanghai Co. Ltd., Shanghai, China) and calibrated with PMMA standards (TOSOH, TOSOH Bioscience Shanghai Co. Ltd., Shanghai, China). ^1^H NMR spectrum was measured by a Bruker 300 MHz nuclear magnetic resonance (NMR) (Bruker Bioscience Shanghai Co. Ltd., Beijing, China) using tetramethylsilane (TMS) as the internal standard and DMSO as the polymer solvent with a temperature of 25 °C. The FT-IR spectra were obtained via a Nicolet 5700 spectrophotometer (Thermo Fisher Technologies (China) Co., Ltd., Shanghai, China) with a resolution of 4 cm^−1^ from 4000 to 500 cm^−1^ by using KBr pellet technique. Copper elemental analysis was made by inductively coupled plasma (ICP) of Vista MPX (Agilent Technologies (China) Co., Ltd., Beijing, China). The elemental analysis was performed on a Vario EL elemental analysis instrument (Elementar Trading Shanghai Co. Ltd., Shanghai, China).

## 3. Results and Discussion

### 3.1. The Properties of Difunctional Reducing Agent, Cell-SH

**Figure 1:**
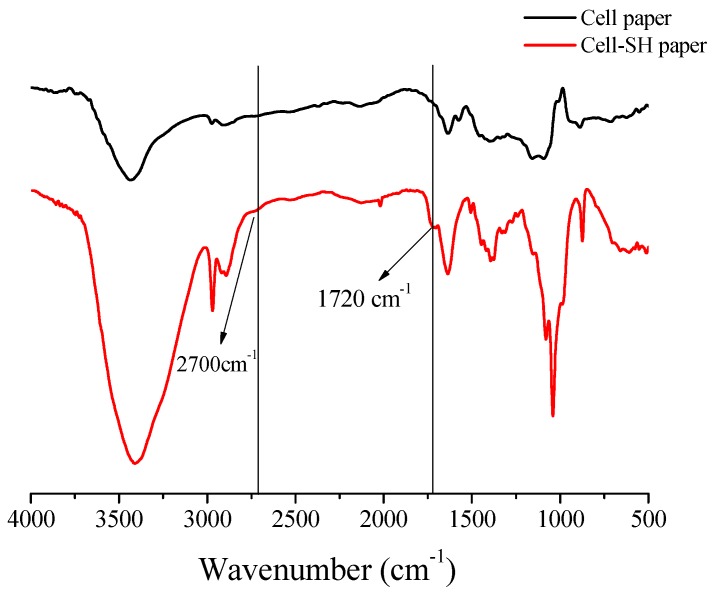
FT-IR spectra of (**a**) pristine cellulose paper; and (**b**) cellulose paper grafted with thioglycolic acid.

The difunctional reducing agent, Cell-SH, was obtained by grafting thioglycolic acid onto the cellulose paper (Scheme 1). Then, the paper was characterized by Elemental Analysis (C: 41.62%, H: 5.23%, S: 7.98%, found from a Vario EL elemental analysis instrument) to estimate the grafting density, which was indicated ca. 0.47 hydroxyl groups were functionalized by thioglycolic acid for each glucose repeat unit. Moreover, the FT-IR spectra of glucose paper before and after functionalized were obtained, which were shown in Figure 1. The characteristic peaks of original paper at 3400, 2900 and 1100 cm^−1^ were corresponding to the stretching vibrations of O–H, C–H and C–O–C bond, respectively. While, the successful graft of thioglycolic acid was confirmed by the appearance of strong band at ca. 1720 cm^−1^ and weak band at ca. 2700 cm^−1^, which was attributable to the carbonyl group of the ester and the sulfhydryl group. The weak band at 660 cm^−1^, corresponding to the C–S stretching vibration, was too weak to be observed. From the characterization above, the thiol group was grafted on the cellulose paper.

### 3.2. The Effect of the Amount of Reducing Agent, Cell-SH and Ligand, PMDETA on the AGET ATRP with Thiol-Functional Cell Paper as Dual Functional Reducing Agent

Reducing agent plays an important role in AGET ATRP. Firstly, we investigated the effect of the amount of reducing agent, Cell-SH on the polymerization, which was shown in Table 1. It’s worth pointing out that the monomer conversion was nearly quantified with proper polymerization time, and the well-defined polymer was obtained (Entry 4 in Table 1). However, the polymerization rate was reduced greatly and the polymerization was out of control without Cell-SH added (Entry 1 in Table 1). From Table 2, the amount of ligand should be more than the copper salt added to establish equilibrium between active radical and dormant species, and the polymerization rate increased with the increase amount of ligand, PMDETA. As discussed above, proper amount of Cell-SH and ligand were necessary to developed AGET ATRP with Cell-SH paper as dual functional role.

### 3.3. Polymerization Kinetics of AGET ATRP of MMA with Cell-SH as Reducing Agent

**Figure 2:**
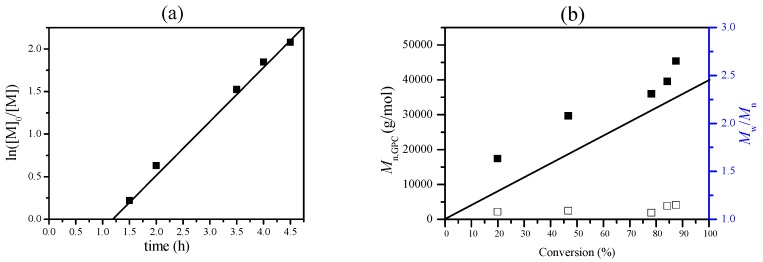
ln([M]_0_/[M]) as a function of polymerization time (**a**); number-average molecular weight (*M*_n,GPC_) and molecular weight distribution (*M*_w_/*M*_n_) versus monomer conversion; (**b**) for AGET ATRP with thiol-functional cellulose paper as reducing agent in the present of limited amount of air (oxygen). Polymerization conditions: [MMA]_0_/[EBPA]_0_/[CuBr_2_]_0_/[PMDETA]_0_ = 400/1/0.25/0.5, *m*_[Cell-SH]_ = 50 mg, *V*_MMA_ = 2.0 mL, *V*_anisole_ = 1.0 mL, Temperature = 90 °C.

In order to further investigated the polymerization behavior, polymerization kinetics of heterogeneous AGET ATRP of MMA in the present of a limited amount of air with Cell-SH paper as reducing agent was conduct and shown in Figure 2. Figure 2a showed the linear relationship between polymerization time and the value of ln([M]_0_/[M]), which indicated that the propagating radical kept constant during the polymerization process, and the polymerization was a first order reaction. In addition, ca. 1.2 h induction period was observed, which demonstrated that a certain time needed to establish dynamic equipment between active radical and dormant species. As shown in Figure 2b, the value of the molecular weight (*M*_n,GPC_) increased with the monomer conversion throughout the polymerization process, meanwhile, the obtained polymer owned narrow molecular weight distributions (*M*_w_/*M*_n_ < 1.2). The results presented above indicated the “living” feature of the AGET ATRP with Cell-SH paper as reducing agent.

### 3.4. Analysis of Chain End and Chain Extension

**Figure 3:**
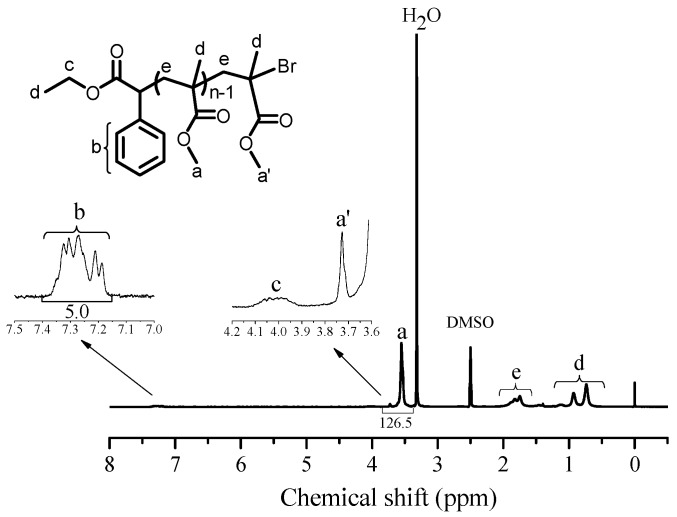
^1^H NMR spectrum of PMMA (*M*_n,GPC_ = 4200 g·mol^–1^, *M*_n,NMR_ = 4300 g·mol^–1^, *M*_w_/*M*_n_ = 1.12) obtained from AGET ATRP using thiol-functional cell paper as reducing agent with DMSO as the solvent and tetramethyl silane (TMS) as an internal standard. Polymerization conditions: [MMA]_0_/[EBPA]_0_/[CuBr_2_]_0_/[PMDETA]_0_ = 400/4/0.25/0.5, *m*_(Cell-SH)_ = 50 mg, *V*_MMA_ = 2.0 mL, *V*_anisole_ = 1.0 mL, Temperature = 90 °C, time = 1.5 h.

To further affirm the “living” character of the novel system, the obtained PMMA by heterogeneous AGET ATRP with Cell-SH paper as reducing agent was analyzed by ^1^H NMR spectroscopy, which was shown in Figure 3. The chemical shifts at 7.15–7.40 ppm (in Figure 3b) and 3.90–4.10 ppm (Figure 3c) belonged to the aromatic protons and methylene ester protons of the initiator, ethyl-2-bromo-2-phenyl acetate (EBPA), which indicated the moieties of EBPA attached to PMMA chain end (α chain end), and EBPA initiated the monomer successfully. The chemical shift at 3.72 ppm (Figure 3a) attributed to the methyl ester protons at the side chain, which deviated from the methyl ester protons in PMMA at 3.6 ppm (Figure 3a), becaused of the electron-withdrawing effect of the ω-Br atom, indicated that the ω chain end of PMMA was end-capped by bromine atom. Moreover, the value of NMR molecular weight (*M*_n,NMR_) calculated from the integrals of peak b, peak a and peak a’ was 4300 g·mol^−1^, which was very closed to the value of GPC molecular weight (*M*_n,GPC_), that is to say, each controlled PMMA chain initiated by EBPA with molar ratio of 1:1, and end fidelity was almost quantitatively.

**Figure 4:**
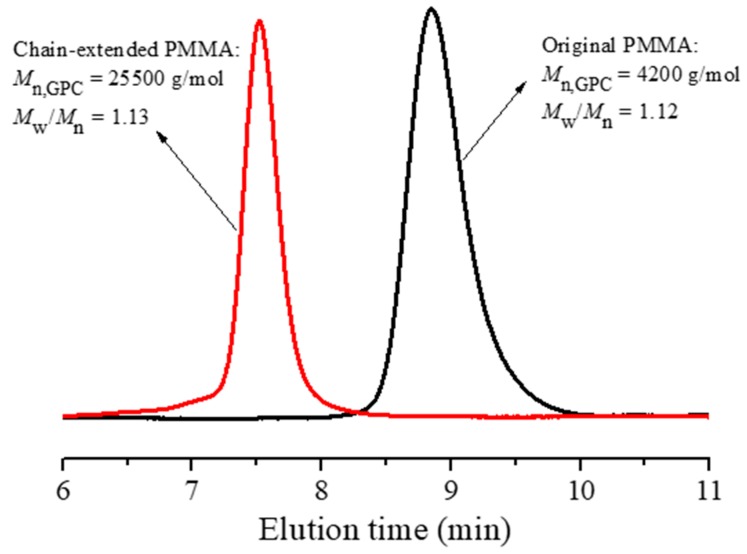
Gel Permeation Chromatography (GPC) curves before and after chain extension using PMMA as the macroinitiator via AGET ATRP with thiol-functional cell paper as reducing agent. Polymerization conditions: original PMMA: [MMA]_0_/[EBPA]_0_/[CuBr_2_]_0_/[PMDETA]_0_ = 400/4/0.25/0.5, *m*_(Cell-SH)_ = 50 mg, *V*_MMA_ = 2.0 mL, *V*_anisole_ = 1.0 mL, Temperature = 90 °C, time = 1.5 h. Chain-extended PMMA: [MMA]_0_/[PMMA-Br]_0_/[CuBr_2_]_0_/[PMDETA]_0_ = 400/2/0.25/0.5, *m*_(Cell-SH)_ = 50 mg, *V*_MMA_ = 1.6 mL, *V*_anisole_ = 1.0 mL, Temperature = 90 °C, time = 2.5 h.

In addition, the obtained PMMA should be employed as a macroinitiator in chain extension reaction. As shown in Figure 4, there was an obvious shift of GPC curves from original controlled PMMA (*M*_n,GPC_ = 4200 g·mol^−1^, *M*_w_/*M*_n_ = 1.12) to chain-extended controlled PMMA with *M*_n,GPC_ = 25,500 g·mol^−1^, *M*_w_/*M*_n_ = 1.13. The successful chain-extension further verified the “living” nature of heterogeneous AGET ATRP with Cell-SH as reducing agent.

### 3.5. Synchronous Separation of Metal Catalyst and Byproduct of Reducing Agent

**Figure 5:**
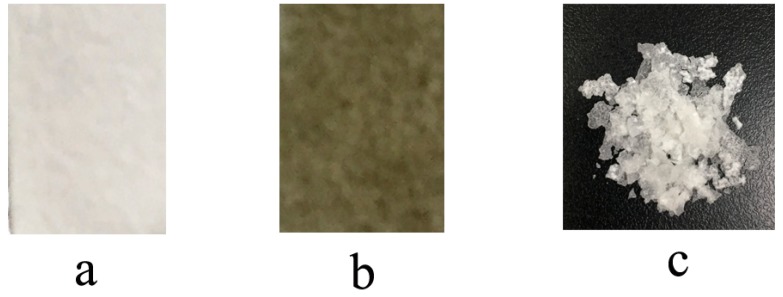
Photographs of (**a**) Cell-SH paper before used as reducing agent; (**b**) Cell-SH paper after used as reducing agent; and (**c**) final PMMA obtained from hetrogeneous AGET ATRP with Cell-SH as reducing agent in the present of a limited of air. Polymerization conditions: [MMA]_0_/[EBPA]_0_/[CuBr_2_]_0_/[PMDETA]_0_ = 400/1/0.25/0.5, *m*_[Cell-SH]_ = 50 mg.

**Figure 6:**
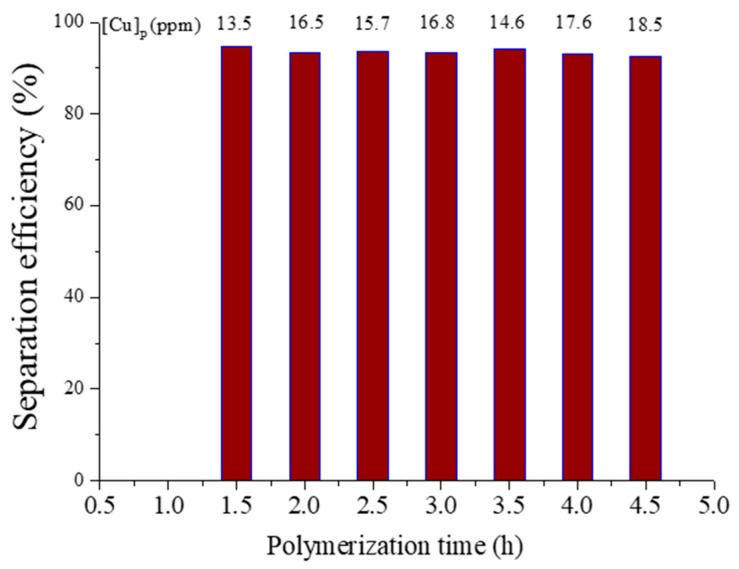
Effect of polymerization time on separation efficiency of the copper catalyst. Polymerization conditions: [MMA]_0_/[EBPA]_0_/[CuBr_2_]_0_/[PMDETA]_0_ = 400/1/0.25/0.5, *m*_[Cell-SH]_ = 50 mg, V_MMA_ = 2.0 mL, V_anisole_ = 1.0 mL, Temperature = 90 °C. [Cu]_P_: the copper residual concentration in obtain polymer.

Heterogeneous AGET ATRP of MMA with Cell-SH paper as reducing agent showed high catalytic activity, outstanding “living” feature as mentioned above. Moreover, the metal catalyst and by product of reducing agent in the AGET ATRP system could be separated synchronously and efficiently. Photographs of the Cell-SH paper before (Figure 5a) and after (Figure 5b) used as reducing agent, and the resultant clean and well-defined PMMA (Figure 5c) were shown in Figure 5. The thiol grafted on cellulose paper can efficiently reduce Cu(II) complex to Cu(I) complex to promote polymerization, accompanied by oxidized to disulfide compounds, which was inclined to adsorb the copper on it. After the polymerization, the metal catalyst adsorbed on paper and byproduct of reducing agent immobilized in the paper could be separated easily and efficiently by filtration. Moreover, the efficient adsorption of copper onto cellulose paper could be visual observed by the change of color (From nearly white (Figure 5a) to dark black (Figure 5b)). In addition, the copper separation efficiencies were calculated from the values of copper residual concentrations (determined by ICP) in the final well-defined polymers, obtained by different polymerization time, which was shown in Figure 6. From Figure 6, the separation efficiencies were higher than 92%, namely nearly perfect removability of the catalyst, which is also observed from the photograph of final PMMA obtained from AGET ATRP using Cell-SH paper as reducing agent (Figure 5c).

## 4. Conclusions

Highly efficient copper catalyst and byproduct of reducing agent synchronous separation was achieved in AGET ATRP with thiol functionalized cellulose paper as dual roles in the presence of a limited amount of air (oxygen). In addition, the CuBr_2_ was employed as metal salt, PMDETA was optimized as ligand, MMA was chosen as model monomer, and EBPA was selected as initiator. The polymerization showed good controllability over molecular weights and molecular weight distributions (*M*_w_/*M*_n_ < 1.2). It is important that the metal catalyst and byproduct of reducing agent could be almost quantitatively separated (>92%) just by filtration the sheet-like reducing agent, and the concentration of residual copper catalyst in obtained PMMA was less than 20 ppm. Therefore, the interesting process may pave a facile way to obtain clean and controlled polymers with different topologies in situ.

## Figures and Tables

**Table 1:** Effect of the amount of Cell-SH on the AGET ATRP with thiol-functional cell paper as the reducing agent.

Entry	*m*_(Cell-SH)_ (mg)	Time (h)	Conv. (%)	*M*_n,th_ (g·mol^−1^)	*M*_n,GPC_ (g·mol^−1^)	*M*_w_/*M*_n_
1	0	17	25.9	10,400	89,000	2.11
2	25	2	28.8	11,500	23,200	1.08
3	50	2	32.2	12,300	27,900	1.12
4	50	17	>99	40,000	56,900	1.28

Polymerization conditions: X = [MMA]_0_/[EBPA]_0_/[CuBr_2_]_0_/[PMDETA]_0_ = 400/1/0.25/0.5, *m*_(Cell-SH)_ = 50 mg, Temperature = 90 °C, *V*_MMA_ = 2.0 mL, *V*_anisole_ = 1.0 mL.

**Table 2:** Effect of the amount of ligand, *N,N,N*′*,N″,N″*-Pentamethyldiethylenetriamine (PMDETA) on the AGET ATRP with thiol-functional cell paper as the reducing agent.

Entry	Volume (mL)	X	Conv. (%)	*M*_n,th_ (g·mol^−1^)	*M*_n,GPC_ (g·mol^−1^)	*M*_w_/*M*_n_
1	4.9	400/1/0.5/0.5	0	NA	NA	NA
2	9.8	400/1/0.5/1	45.9	18,400	25,500	1.09
3	14.7	400/1/0.5/1.5	70.2	28,100	39,000	1.25
4	19.6	400/1/0.5/2	78.2	31,300	45,200	1.28

Polymerization conditions: X = [MMA]_0_/[EBPA]_0_/[CuBr_2_]_0_/[PMDETA]_0_, *m*_(Cell-SH)_ = 50 mg, *V*_MMA_ = 2.0 mL, *V*_anisole_ = 1.0 mL, Temperature = 90 °C, time = 3.5 h.

## References

[1] Matyjaszewski K., Tsarevsky N.V. (2014). Macromolecular Engineering by Atom Transfer Radical Polymerization. J. Am. Chem. Soc..

[2] Ouchi M., Sawamoto M. (2017). 50th Anniversary Perspective: Metal-Catalyzed Living Radical Polymerization: Discovery and Perspective. Macromolecules.

[3] Matyjaszewski K. (2012). Atom Transfer Radical Polymerization (ATRP): Current Status and Future Perspectives. Macromolecules.

[4] D’hooge D.R., Van Steenberge P.H.M., Reyniers M.-F., Marin G.B. (2016). The strength of multi-scale modeling to unveil the complexity of radical polymerization. Prog. Polym. Sci..

[5] Van Steenberge P.H.M., D’hooge D.R., Wang Y., Zhong M., Reyniers M.-F., Konkolewicz D., Matyjaszewski K., Marin G.B. (2012). Linear Gradient Quality of ATRP Copolymers. Macromolecules.

[6] Kamigaito M., Ando T., Sawamoto M. (2001). Metal-catalyzed living radical polymerization. Chem. Rev..

[7] Wang J.-S., Matyjaszewski K. (1995). Controlled/“living” radical polymerization. Atom transfer radical polymerization in the presence of transition-metal complexes. J. Am. Chem. Soc..

[8] Matyjaszewski K., Xia J. (2001). Atom Transfer Radical Polymerization. Chem. Rev..

[9] Konkolewicz D., Magenau A.J.D., Averick S.E., Simakova A., He H., Matyjaszewski K. (2012). ICAR ATRP with ppm Cu Catalyst in Water. Macromolecules.

[10] Bai L., Zhang L., Cheng Z., Zhu X. (2012). Activators generated by electron transfer for atom transfer radical polymerization: Recent advances in catalyst and polymer chemistry. Polym. Chem..

[11] Jakubowski W., Matyjaszewski K. (2005). Activator Generated by Electron Transfer for Atom Transfer Radical Polymerization. Macromolecules.

[12] Payne K.A., Van Steenberge P.H.M., D’Hooge D.R., Reyniers M.-F., Marin G.B., Hutchinson R.A., Cunningham M.F. (2014). Controlled synthesis of poly[(butyl methacrylate)-*co*-(butyl acrylate)] via activator regenerated by electron transfer atom transfer radical polymerization: Insights and improvement. Polym. Int..

[13] Payne K.A., D’hooge D.R., Van Steenberge P.H.M., Reyniers M.-F., Cunningham M.F., Hutchinson R.A., Marin G.B. (2013). ARGET ATRP of Butyl Methacrylate: Utilizing Kinetic Modeling To Understand Experimental Trends. Macromolecules.

[14] Fierens S., D’Hooge D., Van Steenberge P., Reyniers M.-F., Marin G. (2015). Exploring the Full Potential of Reversible Deactivation Radical Polymerization Using Pareto-Optimal Fronts. Polymers.

[15] Matyjaszewski K., Jakubowski W., Min K., Tang W., Huang J., Braunecker W.A., Tsarevsky N.V. (2006). Diminishing catalyst concentration in atom transfer radical polymerization with reducing agents. Proc. Natl. Acad. Sci. USA.

[16] Jiang X.W., Wu J., Zhang L.F., Cheng Z.P., Zhu X.L. (2014). Highly Active ppm Level Organic Copper Catalyzed Photo-Induced ICAR ATRP of Methyl Methacrylate. Macromol. Rapid Commun..

[17] Zhu G., Zhang L., Zhang Z., Zhu J., Tu Y., Cheng Z., Zhu X. (2011). Iron-Mediated ICAR ATRP of Methyl Methacrylate. Macromolecules.

[18] Kwak Y., Magenau A.J.D., Matyjaszewski K. (2011). ARGET ATRP of Methyl Acrylate with Inexpensive Ligands and ppm Concentrations of Catalyst. Macromolecules.

[19] Shen Y., Zhu S., Pelton R. (2001). Effect of ligand spacer on silica gel supported atom transfer radical polymerization of methyl methacrylate. Macromolecules.

[20] Faucher S., Zhu S. (2007). Fundamentals and development of high-efficiency supported catalyst systems for atom transfer radical polymerization. J. Polym. Sci. Part A Polym. Chem..

[21] Ding S., Xing Y., Radosz M., Shen Y. (2006). Magnetic nanoparticle supported catalyst for atom transfer radical polymerization. Macromolecules.

[22] Karimi B., Zamani A., Clark J.H. (2005). A Bipyridyl Palladium Complex Covalently Anchored onto Silica as an Effective and Recoverable Interphase Catalyst for the Aerobic Oxidation of Alcohols. Organometallics.

[23] Shen Y.Q., Tang H.D., Ding S.J. (2004). Catalyst separation in atom transfer radical polymerization. Prog. Polym. Sci..

[24] Faucher S., Zhu S. (2004). Location of the Catalytic Site in Supported Atom Transfer Radical Polymerization. Macromol. Rapid Commun..

[25] Shen Y., Zhu S., Pelton R. (2001). Soluble and recoverable support for copper bromide-mediated living radical polymerization. Macromolecules.

[26] Hong S.C., Lutz J.-F., Inoue Y., Strissel C., Nuyken O., Matyjaszewski K. (2003). Use of an Immobilized/Soluble Hybrid ATRP Catalyst System for the Preparation of Block Copolymers, Random Copolymers, and Polymers with High Degree of Chain End Functionality. Macromolecules.

[27] Yang J., Ding S., Radosz M., Shen Y. (2004). Reversible catalyst supporting via hydrogen-bonding-mediated self-assembly for atom transfer radical polymerization of MMA. Macromolecules.

[28] Du X., Pan J., Chen M., Zhang L., Cheng Z., Zhu X. (2014). Thermo-regulated phase separable catalysis (TPSC)-based atom transfer radical polymerization in a thermo-regulated ionic liquid. Chem. Commun..

[29] Ding S., Radosz M., Shen Y. (2005). Ionic Liquid Catalyst for Biphasic Atom Transfer Radical Polymerization of Methyl Methacrylate. Macromolecules.

[30] Haddleton D.M., Jackson S.G., Bon S.A.F. (2000). Copper(I)-Mediated Living Radical Polymerization under Fluorous Biphasic Conditions. J. Am. Chem. Soc..

[31] Bai L., Zhang L., Pan J., Zhu J., Cheng Z., Zhu X. (2013). Developing a Synthetic Approach with Thermoregulated Phase-Transfer Catalysis: Facile Access to Metal-Mediated Living Radical Polymerization of Methyl Methacrylate in Aqueous/Organic Biphasic System. Macromolecules.

[32] Sarbu T., Pintauer T., McKenzie B., Matyjaszewski K. (2002). Atom transfer radical polymerization of styrene in toluene/water mixtures. J. Polym. Sci. Part A Polym. Chem..

[33] Pan J., Zhang L., Bai L., Zhang Z., Chen H., Cheng Z., Zhu X. (2013). Atom transfer radical polymerization of methyl methacrylate with a thermo-responsive ligand: Construction of thermoregulated phase-transfer catalysis in an aqueous-organic biphasic system. Polym. Chem..

[34] Jiang X.W., Zhang L.F., Cheng Z.P., Zhu X.L. (2016). Highly Efficient and Facile Photocatalytic Recycling System Suitable for ICAR ATRP of Hydrophilic Monomers. Macromol. Rapid Commun..

[35] Jiang X.W., Wu J., Zhang L.F., Cheng Z.P., Zhu X.L. (2016). A Facile Strategy for Catalyst Separation and Recycling Suitable for ATRP of Hydrophilic Monomers Using a Macroligand. Macromol. Rapid Commun..

[36] Miyake G.M., Theriot J.C. (2014). Perylene as an Organic Photocatalyst for the Radical Polymerization of Functionalized Vinyl Monomers through Oxidative Quenching with Alkyl Bromides and Visible Light. Macromolecules.

[37] Theriot J.C., Lim C.-H., Yang H., Ryan M.D., Musgrave C.B., Miyake G.M. (2016). Organocatalyzed atom transfer radical polymerization driven by visible light. Science.

[38] Pan X., Fang C., Fantin M., Malhotra N., So W.Y., Peteanu L.A., Isse A.A., Gennaro A., Liu P., Matyjaszewskit K. (2016). Mechanism of Photoinduced Metal-Free Atom Transfer Radical Polymerization: Experimental and Computational Studies. J. Am. Chem. Soc..

[39] Liu X., Zhang L., Cheng Z., Zhu X. (2016). Metal-free photoinduced electron transfer-atom transfer radical polymerization (PET-ATRP) via a visible light organic photocatalyst. Polym. Chem..

[40] Treat N.J., Sprafke H., Kramer J.W., Clark P.G., Barton B.E., de Alaniz J.R., Fors B.P., Hawker C.J. (2014). Metal-Free Atom Transfer Radical Polymerization. J. Am. Chem. Soc..

[41] Liu P.-Y., Jiang N., Zhang J., Wei X., Lin H.-H., Yu X.-Q. (2006). The Oxidative Damage of Plasmid DNA by Ascorbic Acid Derivatives in vitro: The First Research on the Relationship between the Structure of Ascorbic Acid and the Oxidative Damage of Plasmid DNA. Chem. Biodivers..

[42] Rull-Barrull J., D’Halluin M., Le Grognec E., Felpin F.X. (2016). A paper-based biomimetic device for the reduction of Cu(II) to Cu(I)–application to the sensing of Cu(II). Chem. Commun..

[43] Rull-Barrull J., D’Halluin M., Le Grognec E., Felpin F.X. (2016). Harnessing the Dual Properties of Thiol-Grafted Cellulose Paper for Click Reactions: A Powerful Reducing Agent and Adsorbent for Cu. Angew. Chem. Int. Ed..

